# Enhancing Postoperative Evaluation of Presbyopia Corrections: Correlation of Visual Curve Indices with Vision-Related Quality of Life

**DOI:** 10.3390/jcm14207149

**Published:** 2025-10-10

**Authors:** Georgios Labiris, Christos Panagis, Christina Mitsi, Eirini-Kanella Panagiotopoulou, Eleftheria Vorgiazidou, Konstantinos K. Delibasis, Minas Bakirtzis

**Affiliations:** 1 Department of Ophthalmology, University Hospital of Alexandroupolis, Dragana, 68100 Alexandoupolis, Greece; glampiri@med.duth.gr (G.L.);; 2 Department of Computer Science and Biomedical Informatics, University of Thessaly, 35100 Lamia, Greecekdelibasis@gmail.com (K.K.D.); 3 Postgraduate Program Study “Medical Engineering and Informatics”, Medical School, Aristotle University of Thessaloniki, 54124 Thessaloniki, Greece

**Keywords:** presbyopia, area-of-the-curve, NEI-VFQ, DDART, visual curve

## Abstract

**Background/Objectives:** The aim of this study was to evaluate the correlation between the visual curve (ViC) and Area of the Curve (AoC) indices and the subjective perception of vision-related quality of life in patients who had undergone pseudophakic presbyopia correction. The central hypothesis was that AoC indices would show stronger correlations with vision-specific quality-of-life measures than single-point visual acuity (VA) assessments. **Methods**: A total of 100 patients who underwent bilateral pseudophakic presbyopia correction at the University Hospital of Alexandroupolis, Greece, were included in the study. Six months following surgery, visual acuity was assessed at nine distances using the DDART tool. The AoC was calculated using VA data from four, five, six, and nine distances, and further categorized into Near Vision AoC (AoCN) and Distance Vision AoC (AoCD). Participants also completed the NEI-VFQ-25 questionnaire to evaluate their subjective vision-related quality of life. **Results**: Statistically significant correlations were observed between AoC values and NEI-VFQ-25 total scores (r = 0.668–0.682, *p* < 0.001), near activity subscale scores (r = 0.656–0.686, *p* < 0.001), and distance activity subscale scores (r = 0.733–0.758, *p* < 0.001). In all analyses, the AoC indices derived from ViC demonstrated stronger correlations with quality-of-life scores than those observed with AoC-derived DCT and single VA measurements, even when the AoC was computed using only four measurement points. **Conclusions**: The AoC metric is a superior indicator of vision-specific quality of life compared to isolated VA measurements. AoC effectively captures the multifaceted nature of functional vision following presbyopia correction.

## 1. Introduction

Presbyopic corrections have increased significantly over the past few years. Several pseudophakic and laser-assisted surgical options have been developed with impressive visual outcomes. Indeed, modern lifestyles command the highest possible visual capacity, even for the middle-aged and senior populations, in order to address the complex daily activities that require optimal distance, intermediate, and near visual acuity (VA) [1,2,3,4,5,6].

However, the advanced presbyopia corrections require advanced methods for the evaluation of the postoperative outcomes since it is well known that the conventional measurement of near and distance VA does not accurately reflect the patient’s visual capacity [7,8,9]. Therefore, a series of contemporary indices have been introduced for the evaluation of the presbyopia corrections; among them, the visual curve (ViC) and the Area of the Curve (AoC) attempt to provide better insight on the efficacy of the surgical interventions [8,10,11].

Unfortunately, the assessment of the aforementioned indices is not an easy task. Defocus curve testing (DCT), which is still considered the gold standard in ViC and AoC assessment, is an extremely demanding process, requiring at least nine VA measurements and manual input of the outcomes to an external mathematical program, while at the same time, it does not address the pupil response and the convergence effect. Therefore, it is no surprise that DCT has never been introduced as a regular test in clinical settings; rather, it serves a niche proportion of presbyopia research trials [11,12,13,14].

The growing emphasis on patient-centered outcomes in ophthalmology has underscored the limitations of relying exclusively on conventional visual acuity charts when evaluating the efficacy of presbyopia-correcting procedures. Standard high-contrast distance and near acuity charts primarily quantify threshold resolution at fixed distances under ideal testing conditions. However, visual function in everyday life involves a more complex integration of distance, intermediate, and near demands, often under variable contrast and lighting conditions. Patients undergoing premium presbyopia correction are increasingly active professionally and socially and thus demand seamless performance across multiple focal points. This discrepancy between traditional acuity testing and functional visual requirements explains why patients with seemingly excellent Snellen or ETDRS outcomes may still report dissatisfaction, reduced quality of life, or difficulty in tasks such as computer use, night driving, or prolonged near work. Consequently, there has been a shift toward more comprehensive functional metrics that attempt to simulate real-life performance, with visual curves and area-based indices emerging as promising surrogates of holistic visual capacity [7,8,9,10,11].

In parallel, patient-reported outcome measures (PROMs) have gained significant importance in refractive and cataract surgery. Instruments such as the National Eye Institute Visual Functioning Questionnaire 25 (NEI-VFQ-25) capture domains including general vision, near and distance activities, mental health impacts, role limitations, and dependency, thereby complementing objective tests. These subjective measures provide essential insights into how patients perceive their visual status and how surgery influences daily functioning beyond what can be quantified in logMAR units. However, PROMs alone are not sufficient for comprehensive assessment as they are inherently influenced by patient expectations, cultural factors, and psychological adaptation. The integration of objective performance indices, such as AoC, with validated PROMs like the NEI-VFQ-25, offers a more balanced framework for evaluating surgical success. By exploring whether higher AoC values correspond to better subjective visual functioning, clinicians can both refine surgical planning and tailor counseling to align outcomes with patient expectations. Moreover, such a correlation could support the incorporation of simplified digital platforms into clinical workflows, providing an efficient and reproducible way of linking quantitative indices with patient-reported experiences. This integrative approach represents a critical step toward personalized presbyopia correction, ensuring that success is defined not only by clinical measurements but also by meaningful improvements in patients’ everyday lives [8,9,10].

Recently, our group published the validation outcomes of a novel method for presbyopia correction outcomes, proposing a web application (Democritus Digital Acuity and Reading Test—DDART) that requires only four VA measurements in order to plot ViC and calculate AoC [15,16]. Contrary to DCT, which simulates distance with trial lenses, DDART takes actual VA measurements at specific distances using computer vision to monitor the distance of the patient from the screen and calibrate the symbols accordingly [9]. VA measurements automatically update a neural network subsystem, which identifies the best polynomial curve fitting and automatically calculates ViC and AoC, significantly simplifying the overall process.

Regardless of the methods for AoC assessment, the correlation of this index with the subjective perception of the patients regarding their visual capacity is yet to be fully explored. Previous studies have focused mainly on objective indices, such as defocus curves and AoC, without examining how these metrics correspond to patients’ reported visual performance. Among the prevalent tools for the subjective assessment of vision-targeted health status is the NEI-VFQ 25, which has been used in numerous reports of premium presbyopic corrections. NEI-VFQ 25 has been validated in several languages, including Greek [17]. To our knowledge, no previous study has systematically correlated AoC with NEI-VFQ outcomes. Demonstrating such a relationship is clinically relevant, as it may bridge the gap between objective performance metrics and real-world patient satisfaction.

Within this context, the primary objective of this study was to explore the correlation of the AoC index with the subjective perception of vision-related health status in premium presbyopia corrections.

## 2. Materials and Methods

### 2.1. Setting

This was a prospective correlation trial. The study protocol adhered to the tenets of the Declaration of Helsinki, and all participants provided written informed consent. The protocol was approved by the Institutional Review Board of the University Hospital of Alexandroupolis, Greece, where the study was conducted. The international registration number for the study is NCT06260852.

### 2.2. Participants

Patients who underwent uncomplicated bilateral pseudophakic presbyopia surgery were recruited on a consecutive-if-eligible basis. Participants received Panoptix (Alcon, Fort Worth, TX, USA) intraocular lens (IOL), Vivity IOL (Alcon, Fort Worth, TX, USA), and Restor IOL (Alcon, Fort Worth, TX, USA) targeting emmetropia. For all participants, when the preoperative manifest astigmatism was above 1 Diopter (D), the corresponding Panoptix toric or Vivity toric IOL was implanted. Preoperative refractive status of the participants included myopic, hyperopic, and emmetropic patients scheduled for presbyopia correction. Preoperative manifest spherical equivalent (SE) ranged from −5.50 D to +3.00 D, with a mean value of −0.45 ± 2.05 D. The presbyopia correction technique was selected based on the patients’ preoperative clinical evaluation and their specific visual needs, in accordance with daily clinical practice. Patients with former incisional ocular surgery, glaucoma, former refractive surgery, fundus disease, dry eye disease, mental or neurological disorders, or inability to understand study objectives were excluded. Only fully completed NEI-VFQ-25 questionnaires were included in the analysis, and participants with missing responses to any activity subscale were excluded.

### 2.3. Data Collection

Bilateral VA measurements were collected six months after the operation of the second eye using the DDART at nine distances of 25, 28, 33, 40, 50, 66, 100, 200, and 300 cm, as described before [10]. Prior to VA measurements, each participant responded to the self-examination version of the Greek NEI-VFQ-25 in the presence of an independent researcher who had no direct involvement in the study. For all patients, the total score, as well as the scores for near and distance activities, were calculated according to the questionnaire’s instructions.

### 2.4. AoC Estimation

DDART-derived AoC estimation has been described before [15,16]. Briefly, AoC represents the region beneath the interpolated polynomial line on the *XY* plane. To calculate AoC, the integral of the polynomial was calculated between the minimum and maximum x-axis values.

Since logarithm of the Minimum Angle of Resolution (logMAR) values can be negative, AoC was defined in terms of VA measured in Letters. Specifically, the AoC for a ViC modeled by an interpolating polynomial *g*(*x*) was calculated as follows:

$$AoC=\int_{x_{min}}^{x_{max}}g\left(x\right)dx.$$

As published before by our group, DDART’s interpolation subsystem can reliably calculate AoC even with four actual VA measurements at specific distances [16]. However, in order to evaluate the performance of DDART’s interpolation subsystem, AoC was computed using the VA data of optimal subsets of distances, more specifically, (a) four distances at 25, 33, 66, and 300 cm, (b) five distances at 25, 33, 50, 100, and 300 cm, (c) six distances at 25, 28, 33, 50, 100, and 300 cm, and (d) finally, all nine distances (25, 28, 33, 40, 50, 66, 100, 200, and 300 cm). Moreover, in all cases, AoC was subdivided into Near Vision AoC (AoCN), including distances up to 50 cm, and Distance Vision AoC (AoCD) for distances greater than 50 cm.

Finally, conventional DCTs were constructed for all patients, with straight lines connecting each focal point. The total AoC, as well as AoCN and AoCD, were also calculated from these curves.

### 2.5. Statistical Analysis

Data normality was assessed using the Shapiro–Wilk test. According to the null hypothesis of this test, data conforming to a normal distribution will yield *p*-values greater than 0.05. Values *p* ≤ 0.05 indicate non-normal distribution. An a priori power analysis was conducted using a correlation test to determine the required sample size. Assuming an expected effect size of 0.26, an alpha level of 0.05, and a desired statistical power of 0.80, the analysis indicated that a minimum of 81 participants would be necessary. The calculation was performed using G*Power software (version 3.1, Universität Düsseldorf, Düsseldorf, Germany). For normally distributed numerical variables with similar variances and no outliers, the Pearson correlation coefficient was used to compute linear correlations. If these assumptions were not met, the non-parametric Spearman correlation coefficient was applied. Spearman’s method is robust against non-normal distributions and outliers. To identify factors associated with less favorable vision-related QoL (lower NEI-VFQ-25 scores), an exploratory analysis was performed. Firstly, demographic, clinical and optical variables between participants were compared in the lowest quartile versus the remaining cohort using Student’s t-tests or Mann–Whitney U tests for continuous variables and chi-square or Fisher’s exact tests for categorical variables. *p* < 0.05 was considered statistically significant. Statistical analyses were performed using MedCalc version 20.010 (MedCalc Software, Mariakerke, Belgium).

## 3. Results

A total of 100 participants were recruited in this study; among them were 54 men and 46 women. The mean age of the study group was 62 ± 8,87 years. The mean VA at 40 cm was 74.4 ± 7.74 Letters and the mean VA at 300 cm was 85.6 ± 4.01 Letters.

### 3.1. Normality Test

The results indicate that most parameters presented a normal distribution, recording *p*-values greater than 0.05 in the normality test, except for distance activities subscale scores (Table 1).

### 3.2. Correlation Coefficient

To minimize potential biases, correlation coefficients were calculated for each pair of parameters using the appropriate statistical method. We specifically calculated the correlation between patients’ AoCN and AoCD from ViC and conventional DCT with NEI-VFQ near (NA) and distance activities (DA) subscale scores, respectively. Furthermore, correlations were performed between the total AoC derived from ViC and DCT and total NEI-VFQ score.

**Figure 1:**
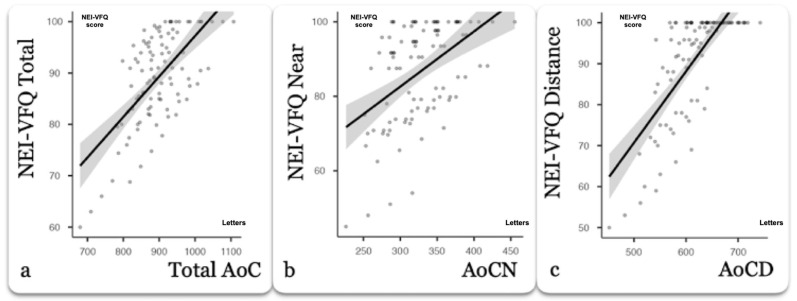
(**a**) Scatterplot of questionnaire score vs. total AoC (Letters), (**b**) scatterplot of questionnaire score vs. AoCN (Letters), and (**c**) scatterplot of questionnaire score vs. AoCD (Letters).

All correlations are presented in Table 2. Total ViC’s AoC demonstrated better correlations with the total NEI-VFQ score from the DCT’s total AoC regardless of the actual VA measurements that were used (all *p* < 0.001). The correlations of AoCN and AoCD derived from ViC with the NEI-VFQ NA and DA scores outperformed the corresponding correlations obtained from single VA measurements and from AoCN and AoCD derived from DCT. The correlation strength was even further improved when AoC was calculated by nine VA measurements for both NA (r = 0.686, *p* < 0.001) and for DA (r = 0.758, *p* < 0.001). Despite that fact, NEI-VFQ correlations with AoC calculated with even four actual VA measurements were robust, as presented in the scatterplots of Figure 1. Single VA measurements at 40 cm and at 300 cm also presented significant correlations with the NA (r = 0.596, *p* < 0.01) and DA (r = 0.621, *p* < 0.001) scores. However, correlation strength was significantly lower than the corresponding AoC scores from both ViC and DCT.

### 3.3. Lower Vision-Related QoL

Post hoc comparison between the lowest NEI-VFQ quartile and the remainder of the cohort (Table 3) showed that participants with a lower score had significantly lower AoC derived from ViC and AoCN and AoCD derived from DCT (Table 4).

There was no statistically significant difference in age, gender distribution, preoperative spherical equivalent, or total AoC derived from DCT between groups (Table 4). These findings suggest that lower objective visual performance (lower AoC) is associated with worse patient-reported visual functioning. Furthermore, the lack of a significant difference in AoC derived from conventional DCT between the two groups indicates that it may be a less reliable predictor of real-life visual performance.

## 4. Discussion

It is well established that premium pseudophakic corrections continuously gain popularity, attempting to restore the pre-presbyopic functionality of the human eye [18]. Major advances in biometry, in intraocular lens technology, in digital-assistance, and in laser-assistance have provided the necessary means to support novel options in presbyopia surgery [19,20,21]. However, despite these advances in surgical planning and techniques, progress in the interpretation of postoperative outcomes has remained relatively limited [11,12].

Among the fundamental reasons for this fact is that presbyopia requires the mathematical calculation and plotting of the whole visual curve of the patient [11,22]. Even today, the gold standard for assessing visual curve is the DCT, an extremely demanding and tiring process, both for the ophthalmologist and the patient. The literature suggests that accurate assessment of the visual curve with DCT requires at least 9 to 12 VA measurements at simulated distances using trial lenses [12,23]. Following VA measurements, all data have to be manually inserted into an advanced mathematical program for the accurate plotting of the visual curve and the calculation of the area of the curve, which actually reflects the overall visual acuity. The complexity of DCT has likely limited its adoption as a routine postoperative test, even in centers that frequently perform premium presbyopia corrections. Therefore, in clinical settings, the vast majority of cataract surgeons rely on the simple measurement of distance and near visual acuity [12,23,24]. Recently, we published the validation outcomes of a novel method for reliable ViC and AoC calculation, based on the DDART application that attempts to simplify the overall process. Briefly, (a) DDART’s computer vision service continuously monitors the examinee’s distance from the screen, (b) the projected symbol’s relative size is automatically adjusted according to the examinee’s distance, (c) VA data are automatically being processed by a neural network subsystem which identifies the optimal polynomial curve fitting, and (d) following optimal curve fitting, ViC is plotted and AoC is automatically calculated, even with only four available VA measurements at specific distances [10,16].

However, regardless of the means of ViC plotting and AoC calculation, their correlation with the patient’s subjective perception of vision-specific quality of life was yet to be explored, and that was the primary objective of present study. To address this, we selected the NEI-VFQ-25, which is the most prevalent relevant instrument used in numerous QoL studies [17]. The objectives of the study were (a) to explore whether fewer actual VA measurements reduced the correlation of the calculated AoC with NEI-VFQ scores and (b) to examine whether AoC derived from ViC demonstrates stronger correlations with subjective measures of vision-related postoperative functional capacity compared with VA and AoC derived from DCT.

Therefore, ViC was plotted and AoC was calculated, allowing the DDART’s neural network subsystem to use VA measurements from four, five, six, and nine distances, and correlations were made in all four cases. Moreover, total AoC was subdivided into Distance and Near Vision AoCs, and correlations were attempted with the NEI-VFQ distance activities and near activities subscale scores, respectively.

In our study, significant correlations were observed between the total AoC derived from ViC and the total NEI-VFQ score, ranging from ρ = 0.668 to 0.686. Stronger correlations were observed between the AoCD and AoCN subscale scores from ViC and the corresponding NEI-VFQ subscale scores (DA: r = 0.733 to 0.758, NA: ρ = 0.656 to 0.686). The correlation between the AoC and NEI-VFQ improved as more actual VA measurements were included for the AoC calculation by DDART; however, even with four VA measurements, we observed a significant correlation. In all cases, the AoC derived from ViC demonstrated a stronger correlation with NEI-VFQ compared to AoC derived from DCT and single VA measurements (DA: r = 0.734, r = 0.621; NA: ρ = 0.652, ρ = 0.596).

In the exploratory analysis, patients belonging to the lowest quartile of NEI-VFQ scores demonstrated a significantly reduced AoC derived from ViC compared with the remainder of the cohort, whereas no significant differences were observed for age, gender, preoperative spherical equivalent, or AoC derived from DCT. These findings indicate that the diminished objective functional performance, as captured by ViC-derived AoC, is associated with poorer patient-reported visual functioning. Importantly, the lack of significant differences in DCT-derived AoC between groups suggests that conventional DCT may be less sensitive in reflecting real-life visual capacity, reinforcing the potential clinical value of ViC-based indices as more reliable predictors of subjective outcomes.

The range of preoperative refractive status in our cohort, spanning from moderate myopia to mild hyperopia, reflects the diversity of patients undergoing presbyopia correction in clinical practice. Importantly, our findings suggest that the preoperative refractive status was not a determinant of postoperative QoL, as no significant differences in NEI-VFQ scores were observed across real-life visual quality subgroups. Instead, the outcomes appeared to depend more on the achievement of higher AoC values, underscoring that precise refractive targeting and functional curve performance are the key factors driving patient satisfaction rather than the baseline refractive error itself.

The concept of using curve-based metrics like AoC has been explored in prior studies. For instance, Wolffsohn et al. investigated the optimal step size for DCT and proposed the “area-of-focus” metric [12]. Buckhurst et al. further correlated the area-of-focus with subjective visual satisfaction using a 0–5 scale [25]. Other researchers, including Terauchi et al. and Lapid-Gortzak et al., have used similar techniques to evaluate age-related visual outcomes and intermediate vision performance, respectively [26,27]. These studies highlight the growing recognition that single-point measures of VA do not fully capture the complexity of functional vision. Curve-based approaches, in contrast, provide a more comprehensive assessment across different focal distances, allowing clinicians to better understand patients’ performance in everyday visual tasks. Nevertheless, despite these contributions, the relationship between the AoC and standardized patient-reported outcome measures, such as the NEI-VFQ-25, has not been systematically explored, leaving an important gap in linking the objective and subjective measures of visual capacity.

We acknowledge that our exploratory subgroup analysis is limited by sample size for some IOL types and by the observational design. As a result, the absence of an independent association between the IOL model and NEI-VFQ should be interpreted cautiously; larger, prospective, stratified studies are required to determine whether certain IOL designs independently affect patient-reported outcomes beyond their impact on the AoC and residual refractive error.

## 5. Conclusions

To our knowledge, this is the first study to explore AoC’s correlation with NEI-VFQ and prospectively evaluate its capacity as an index of self-perceived, vision-specific quality of life. Since AoC provides robust correlations with NEI-VFQ, even with four actual VA measurements, we are confident that our study outcomes will motivate cataract surgeons who practice premium corrections to measure AoC in their daily clinical practice. Modern applications like the DDART simplify the ViC and AoC assessment and provide better insight on the efficacy of presbyopia interventions. Therefore, upon confirmation of our study outcomes by other cataract surgeons, ViC and AoC might substitute single VA measurements, even in clinical settings.

## Figures and Tables

**Table 1:** Normality distribution results.

Feature	*p*-Value
Total ViC’s AoC	0.7608
ViC’s AoCN	0.5349
ViC’s AoCD	0.9086
Total DCT’s AoC	0.261
DCT’s AoCN	0.183
DCT’s AoCD	0.465
NEI-VFQ 25 Score	0.3555
Near Activities Score	0.1685
Distance Activities Score	0.0051
VA at 40 cm	0.9423
VA at 300 cm	0.1726

VA: visual acuity, AoC: Area of the Curve, AoCN: Near Area of the Curve, AoCD: Distance Area of the Curve, ViC: visual curve, DCT: Defocus Curves Testing, and NEI-VFQ: National Eye Institute Visual Functioning Questionnaire.

**Table 2:** Correlation coefficient results between NEI-VFQ and AoC generated using different number of VA measurements.

AoC	Num. of VA Actual Measurements for AoC Calculation	NEI-VFQ 25	Correlation Method	Correlation Coefficient [95% CI]
Total AoC	4	Total Score	Pearson	0.668 *** [0.471, 0.841]
	5		Pearson	0.670 *** [0.473, 0.842]
	6		Pearson	0.684 *** [0.496, 0.849]
	9		Pearson	0.682 *** [0.539, 0.763]
	Conventional DCT		Pearson	0.664 *** [0.38, 0.834]
AoCN	4	Near Activities Score	Pearson	0.686 *** 0.399, 0.850]
	5		Pearson	0.686 *** [0.399, 0.851]
	6		Pearson	0.656 *** [0.355, 0.836]
	9		Pearson	0.686 *** [0.486, 0.868]
	Conventional DCT		Pearson	0.652 *** [0.362, 0.827]
	1 (40 cm)		Pearson	0.596 ** [0.389, 0.737]
AoCD	4	Distance Activities Score	Spearman	0.733 *** [0.535, 0.892]
	5		Spearman	0.753 *** [0.645, 0.922]
	6		Spearman	0.744 *** [0.639,0.921]
	9		Spearman	0.758 *** [0.735, 0.952]
	Conventional DCT		Spearman	0.734 *** [0.669, 0.923]
	1 (300 cm)		Spearman	0.621 *** [0.642, 0.916]

VA: visual acuity, AoC: Area of the Curve, AoCN: Near Vision Area of the Curve, AoCD: Distance Vision Area of the Curve, CI: Confidence Interval, DCT: Defocus Curves Testing, ** *p* < 0.01, and *** *p* < 0.001.

**Table 3:** Mean values of NEI-VFQ’s lowest scores.

	Group	Mean ± SD
NEI-VFQ Total	Lowest quartile	76.7 ± 6.69
	Highest quartiles	94 ± 5.21
NEI-VFQ Near	Lowest quartile	70.8 ± 8.96
	Highest quartiles	93.5 ± 7.31
NEI-VFQ Distance	Lowest quartile	73 ± 8.96
	Highest quartiles	93.5 ± 7.31

**Table 4:** Comparison of NEI-VFQ scores between lowest and highest quartiles of visual function indices.

Indices	NEI-VFQ Scores	Mean ± SD	*p* Value
Total AoC 9 (Letters/cm)	Lowest quartile	830 ± 63.9	<0.001 ***
	Highest quartiles	917 ± 63.0	
Total AoC 4 (Letters/cm)	Lowest quartile	824 ± 75.0	<0.001 ***
	Highest quartiles	877 ± 58.4	
Total AoC 5 (Letters/cm)	Lowest quartile	823 ± 77.4	<0.001 ***
	Highest quartiles	876 ± 59.0	
Total AoC 6 (Letters/cm)	Lowest quartile	826 ± 67.6	<0.001 ***
	Highest quartiles	875 ± 52.7	
Conventional Total AoC (Letters/cm)	Lowest quartile	823 ± 71.0	0.06
	Highest quartiles	874 ± 50.7	
AoCN 9 (Letters/cm)	Lowest quartile	286 ± 31.2	<0.001 ***
	Highest quartiles	338 ± 39.9	
AoCN 4 (Letters/cm)	Lowest quartile	269 ± 24.1	<0.001 ***
	Highest quartiles	292 ± 25.7	
AoCN 5 (Letters/cm)	Lowest quartile	267 ± 23.9	<0.001 ***
	Highest quartiles	293 ± 26.0	
AoCN 6 (Letters/cm)	Lowest quartile	271 ± 13.1	<0.001 ***
	Highest quartiles	291 ± 24.1	
Conventional AoCN (Letters/cm)	Lowest quartile	262 ± 17.0	0.002 **
	Highest quartiles	294 ± 24.7	
AoCD 9 (Letters/cm)	Lowest quartile	547 ± 36.7	<0.001 ***
	Highest quartiles	567 ± 33.5	
AoCD 4 (Letters/cm)	Lowest quartile	539 ± 39.1	0.001 **
	Highest quartiles	588 ± 34.6	
AoCD 5(Letters/cm)	Lowest quartile	528 ± 38.1	<0.001 ***
	Highest quartiles	591 ± 36.0	
AoCD 6 (Letters/cm)	Lowest quartile	531 ± 33.5	<0.001 ***
	Highest quartiles	584 ± 30.8	
Conventional AoCD (Letters/cm)	Lowest quartile	535 ± 33.7	<0.001 ***
	Highest quartiles	584 ± 27.5	
Age (Years)	Lowest quartile	66.2 ± 10.2	0.66
	Highest quartiles	60.2 ± 6.73	
Gender	Lowest quartile	NA	0.075
	Highest quartiles	NA	
Preoperative spherical equivalent (D)	Lowest quartile	−0.57 ± 2.02	0.12
	Highest quartiles	−0.34 ± 2.12	

AoC: Area of the Curve, AoCN: Near Vision Area of the Curve, AoCD: Distance Vision Area of the Curve, SD: Standard Deviation, D: Diopters ** *p* < 0.01, and *** *p* < 0.001.

## Data Availability

The data supporting the findings of this study are available from the corresponding author upon reasonable request. Due to ethical and privacy considerations, certain restrictions may apply to the availability of the data.

## Abbreviations

The following abbreviations are used in this manuscript:

VA	Visual Acuity
ViC	Visual Curve
AoC	Area of the Curve
DDART	Democritus Digital Acuity and Reading Test
NEI-VFQ 25	National Eye Institute Visual Functioning Questionnaire 25
IOL	Intraocular Lens
logMAR	Logarithm of the Minimum Angle of Resolution
AoCN	Near Vision Area of the Curve
AoCD	Distance Vision Area of the Curve
NA	Near Activities
DA	Distance Activities
DCT	Defocus Curve Testing
CI	Confidence Interval
PROMs	Patient-Reported Outcome Measures
